# Assessment and Mitigation of Exposure of 3-D Printer Emissions

**DOI:** 10.3389/ftox.2021.817454

**Published:** 2022-02-18

**Authors:** Boowook Kim, Jae Hoo Shin, Hoi Pin Kim, Mi Seong Jo, Hee Sang Kim, Jong Sung Lee, Hong Ku Lee, Hyuk Cheol Kwon, Sung Gu Han, Noeul Kang, Mary Gulumian, Dhimiter Bello, Il Je Yu

**Affiliations:** ^1^ Institute of Health and Environment, Seoul National University, Seoul, Korea; ^2^ Institute of Occupation and Environment, Korea Workers’ Compensation and Welfare Service, Incheon, Korea; ^3^ Aerosol Toxicology Research Center, HCTm, Incheon, Korea; ^4^ Toxicology Laboratory, Sanghuh College of Life Science, Konkuk University, Seoul, Korea; ^5^ Department of Respiratory Medicine, Samsung Hospital, Seoul, Korea; ^6^ Haematology and Molecular Medicine, University of the Witwatersrand, Johannesburg, South Africa; ^7^ Water Research Group, Unit for Environmental Sciences and Management, North West University, Potchefstroom, South Africa; ^8^ Department of Biomedical and Nutritional Sciences, University of Massachusetts, Lowell, MA, United States; ^9^ HCT, Co., Incheon, Korea

**Keywords:** 3-D printer, emission, exposure assessment, mitigation, particles, volatile organic chemicals (VOCs)

## Abstract

This study monitored particulates, and volatile organic compounds (VOCs) emitted from 3-D printers using acrylonitrile-butadiene-styrene copolymer (ABS) filaments at a workplace to assess exposure before and after introducing exposure mitigation measures. Air samples were collected in the printing room and adjacent corridor, and real-time measurements of ultrafine and fine particle were also conducted. Extensive physicochemical characterizations of 3-D printer emissions were performed, including real-time (size distribution, number concentration) nanoparticle characterization, size-fractionated mass distribution and concentration, as well as chemical composition for metals by ICP-MS and VOCs by GC-FID, real-time VOC monitors, and proton-transfer-reaction time-of-flight mass spectrometer (PTR-TOF-MS). Air sampling showed low levels of total suspended particulates (TSP, 9–12.5/m^3^), minimal levels (1.93–4 ppm) of total volatile organic chemicals (TVOC), and formaldehyde (2.5–21.7 ppb). Various harmful gases, such as formaldehyde, acrolein, acetone, hexane, styrene, toluene, and trimethylamine, were detected at concentrations in the 1–100 ppb by PTR-TOF-MS when air sample was collected into the Tedlar bag from the front of the 3-D printer. Ultrafine particles having an average particle size (30 nm count median diameter and 71 nm mass median diameter) increased during the 3-D printing operation. They decreased to the background level after the 3-D printing operation, while fine particles continually increased after the termination of 3-D printing to the next day morning. The exposure to 3-D printer emissions was greatly reduced after isolating 3-D printers in the enclosed space. Particle number concentration measured by real-time particle counters (DMAS and OPC) were greatly reduced after isolating 3-D printers to the isolated place.

## Introduction

The newly developed technology of 3-D printing, a collective term for additive manufacturing or fused deposition modeling (FFD), is penetrating the marketplace fast and can be found in many teaching laboratories in universities and 1–12 grade schools, as well as in research laboratories and industrial settings. During 3-D printing, objects are manufactured from a computer-assisted design model by successively adding material layer by layer. The composition of the filament used in 3-D printing is modified to meet final product specifications, and increasingly, more filament options are available. Acrylonitrile-butadiene-styrene copolymer (ABS) or Polylactic acid (PLA) filaments are used for fused filament fabrication (FFF) or FFD printing machines. ABS filaments require a higher extruder nozzle to form thermoplastic resins in the solid-state than PLA which melts at a lower temperature (Kim et al., 2015; Azimi et al., 2016; Floyd et al., 2017). ABS printing has been known to emit micro- or nano-sized particles and volatile organic chemicals (VOCs) when ABS is processed and converted to the molten stage. Several publications on particle emission from the 3-D printing reported emissions of ultrafine (or nanoscale) particles (defined as 1–100 nm) (Kim et al., 2015; Azimi et al., 2016; Yi et al., 2016; Floyd et al., 2017; Zhang et al., 2019). Nanoparticle emissions present a health concern for operators of 3-D printers, as well as bystanders/occupants who share the same place. Recently, 3-D printing has been regarded as an advanced material consisting of various substances for new types of manufacturing and processing. Risk assessment and management have been discussed at the OECD and EU levels.

The health effects caused by 3-D printing exposure drew attention in Korea recently, after two teachers who used 3-D printers frequently for teaching science courses in their high schools were diagnosed with sarcoma. Moreover, four more teachers in three different high schools were confirmed to have developed cancer, three with sarcomas and one with another type of cancer. 3-D printers were widely distributed and used in more than 50% of these elementary, middle, and high schools (Ohmy News, 2020). Although there is a need for surveillance on schools using 3-D printers, such studies were not possible at this time due to schools closure caused by the new coronavirus pandemic. In response to these health concerns, the current workplace under investigation (a factory manufacturing headphone hangers) took a precautionary. In this study, we attempted to characterize particles and VOC emissions from ABS 3-D printers during the operation and after the introduction of mitigation measures.

## Materials and Methods

### Sampling Sites

**FIGURE 1:**
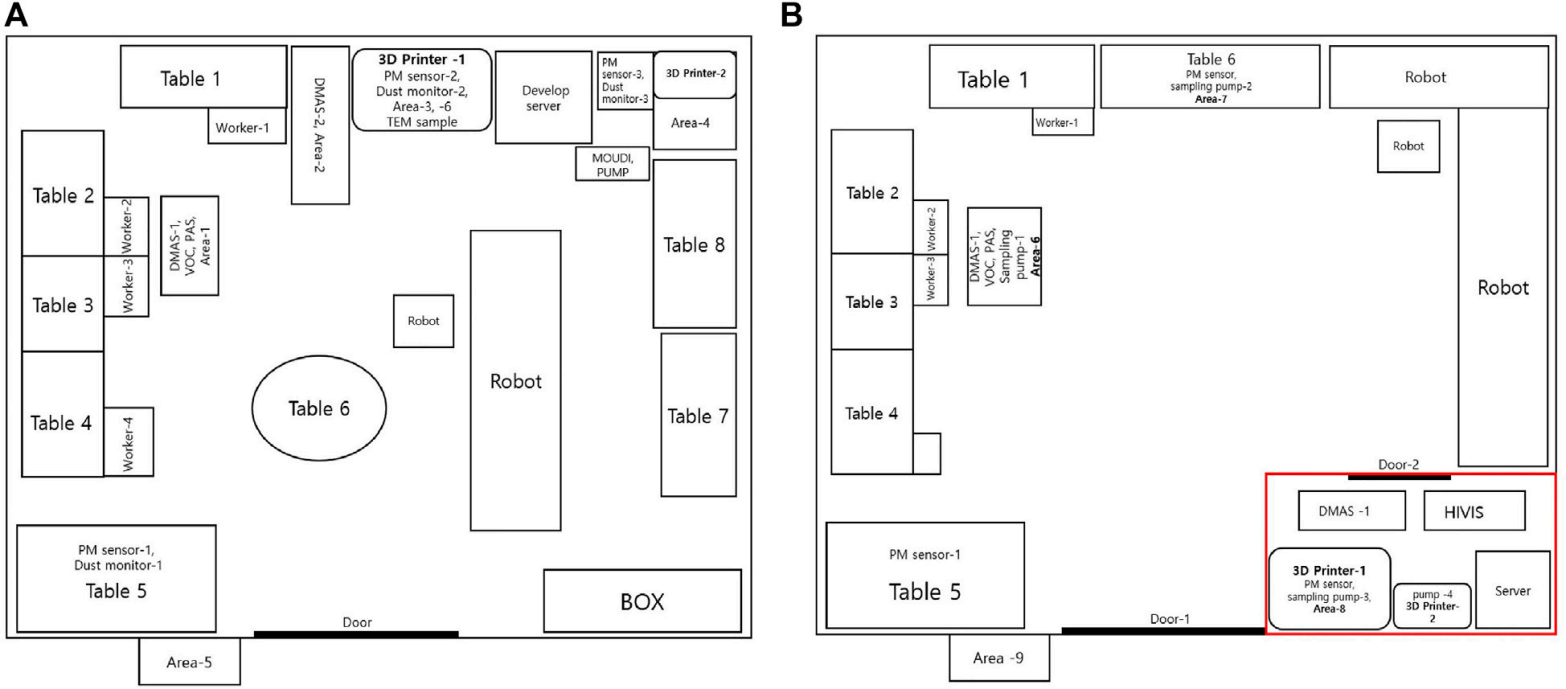
**(A)** Workplace layout after mitigation measure. Mitigation measure dimension (red line): 2.48 m × 1.67 m × 2.72 m. DMAS, Differential mobility analyze system; PAS, photoelectric aerosol sensor; PM sensor, particulate matter sensor. **(B)** Workplace layout, 3-D printer location, and sampling locations. Dimension: 6.75 m × 11.25 m × 2.72 m. DMAS, Differential mobility analyze system; PAS, photoelectric aerosol sensor; PM sensor, particulate matter sensor.

The current study measured the 3-D printer emission particles and VOC concentrations at a workplace equipped with two 3-D printers (Sindoh - 3-DWOX 7X and AFINIA 3-D - H800+) and Universal Robots (UR). In addition, VOC sampling was conducted outside the corridor of the workplace. Figure 1A shows a description of the workplace layout, sampling sites, and other contextual information. Four male workers were located in front of Tables 1–4. The workers were computer programmers involved in developing the facility management system and UR robot operation. The UR robots were not operated on the day of the tests. The dimension of the room was 6.75 m × 11.25 m × 2.72 m (206.55 m^3^). The workplace had only one entrance door and no window and was equipped with three fresh air inlets and three returns located on the ceiling. One ceiling-mounted air conditioner unit was operated during the workday. The manufacturing computer-aided design (CAD) objects consumed 46 and 102 g of ABS filament (containing 95–100% ABS; 0–5% stabilizer; melting point 180–200°C; Plasil, 3Dink Inc, Yangju, Korea) for printers 1 and 2, respectively, to manufacture headphone hangers (Supplementary Figure S1). The sequence of events for a 3-D printing workplace was described in Supplementary Table S1.

### Air Sampling

Figure 1A shows the workplace setup and layout, air sampling sites for area sampling, and real-time monitoring sites. The area air samples were taken by drawing air through polyvinyl chloride (PVC) filters in sampling cassettes (0.45 µm nominal pore size, 37 mm diameter, 2-inch conductive cowl) obtained from Pall Corp (P/N 64,678; Michigan United States). The filter samples for particles were collected from the location described in Figure 1A, and sampling pumps (MSA, Escort Elf pump) were operated at a flow rate of 1.5–2.0 L/min. The sampling was performed during the normal work period from 08:30 to 17:30.

### Real-Time Aerosol Monitoring

Two differential mobility analyzing systems (DMAS) combining differential mobility analyzer (DMA-20, 4220, range 6–225 nm, HCT Co., Ltd. Korea; TSI, United States) and condensation particle counters (CPC, 3775, size range 4 nm- 1, TSI INC., Shoreview, MN 0–108 particles/cm^3^ detection range) were used to monitor the particle size distribution with an electrical mobility diameter ranging from 15 to 710.5 nm. Meanwhile, three types of dust monitors (Model 1.109, range 0.25–32, Grimm, Douglasville, GA; Model PS-1601PM, range 0.25–10, HCTm, Co. Incheon, Korea; PS-1601PMe, range 0.25–10 μm, HCTm, Co. Incheon, Korea) were used to monitor the particle size distribution with a diameter ranging from 0.25 to 32 µm. The workplace air was sampled at a flow rate of 0.3 and 1.2 L/min for the DMAS and dust monitor, respectively. The DMAS scanned the particle sizes at a time resolution of 2.5 min (120 s for up-scan and 30 s for retrace), while the average time for the dust monitor was 1 min. The real-time aerosol monitoring lasted 3 days at the 3-D printing workplace. All the sequences of events that might affect particle monitoring were recorded (Supplementary Table S1).

### Air Sampling for Volatile Organic Chemicals (VOCs) and Monitoring Polycyclic Aromatic Hydrocarbons (PAHs)

Benzene, styrene, and formaldehyde were measured at six locations by area sampling using Coconut Charcoal sorbent tubes (SKC Cat. 226-01) (NIOSH (National Institute of Occupational Safety and Health), 2003). Three grab samples were collected from 8:30–11:50 (200 min), 13:30–16:00 (150 min), and 16:20–17:40 (80 min) to monitor changes in concentration over time and to prevent possible analyte breakthrough from the sorbent tube. For measurement and analysis, benzene and styrene were sampled with a low flow (50 ml/min) air sampling pump (Model LFS-113; Gilian Instrument Corp., West Cladwell, NJ, United States) in SKC Coconut Charcoal sorbent tubes (SKC Cat. 226-01) (Stoehr et al., 2015). The samples were analyzed with a gas chromatography-flame ionization detector (GC-FID). Formaldehyde was analyzed by high-performance liquid chromatography - ultraviolet light detector (HPLC-UV) after flow-through with 2,4-dinitrophenylhydrazine coated silica gel tube (SKC Cat. No. 226-119). The actual sampling flow rate of the pump was calculated as an average value after measuring the flow rate before and after the measurement using a DryCal^®^ primary calibration standard (BIOS, Butler, NJ, United States). The analysis was conducted by the Institute of Occupation and Environment, Korea Workers’ Compensation and Welfare Service, which participates in the American Industrial Hygiene Association (AIHA) Proficiency Analytical Testing (PAT) program. Analysis limit of detection (LOD) was 0.0055 mg/sample for benzene, 0.0088 mg/sample for styrene, and 0.0018 μg/sample for formaldehyde. The real-time TVOC concentration was measured using the EVM-7 Multiparameter Environmental Monitor (Quest Technologies, Inc., United States). The real-time concentration of particle-bound PAH was measured with a real-time photoelectric aerosol sensor PAH 2000 (EcoChem Analytics, League City, TX, United States).

### Proton-Transfer-Reaction Time-Of-Flight Mass Spectrometer (PTR-TOF-MS) Analysis

Since most of the VOCs, evaluated by the NIOSH NMAM 1301 (GC FID) (NIOSH (National Institute of Occupational Safety and Health), 1994a), were not detected, further analysis was performed using a high resolution and high sensitivity PTR-TOF-MS (IONICON Analytik, AUT). When the 3-D printer was running, 10 L of air was collected in the Tedlar bag using a grab air sampling pump (SKC Cat. No. 222–2301) from the front of the printer. The air collected in the Tedlar bag was directly injected into the PTR-TOF-MS to analyze the VOCs.

### Mass Size Distribution Measurement (Mass Median Aerodynamic Diameter, MMAD)

The MMAD of particles generated from the 3-D printer was measured using a foil filter for each stage (diameter, 47 mm; pore size, 5 mm; SKC, Inc., Eighty-Four, PA, United States) with a Nano MOUDI impactor (MOUDI 125 NR, MSP Co., MN, United States) composed of 13 stages (0.01, 0.018, 0.032, 0.056, 0.10, 0.18, 0.32, 0.56, 1.0, 1.8, 3.2, 5.6, and 10 mm). The geometric standard deviation (GSD) for the MMAD was derived from the cumulative mass distribution of the micro-orifice uniform deposition impactor (MOUDI).

### Transmission Electron Microscopy (TEM)

An electrostatic precipitator ESPnano (Model 100; ESPnano, Spokane, WA, United States), operating at the standard sampling flow rate of 0.1 L/min, was used to collect aerosol particles on electron microscopy grids. The TEM nickel grid (Formvar/Carbon 200 mesh, TEDpella, CA, United States) or holey TEM grid (Quantifoil 656-200-Cu; Tedpella, Inc., Redding, CA, United States) were further examined under a transmission electron microscope (TEM, H - 7650; Hitachi, Tokyo, Japan) equipped with an EDX (energy dispersive X-ray analyzer, TM200; Oxford Instruments PLC, Oxfordshire, United Kingdom) at an acceleration voltage of 100 kV (NIOSH (National Institute of Occupational Safety and Health), 1994b).

### Exposure Assessment After Mitigation Measure

After noticing the 3-D printer emission exposure status, the company took an exposure mitigation measure, as shown in Figure 1B. Two 3-D printers were isolated to the enclosed space (which has ventilation on the ceiling). Real-time aerosol monitoring and formaldehyde sampling were conducted inside and outside of the enclosed space.

## Results

### Indoor Air Quality in the Workplace

The workplace average temperature was 26.9°C and ranged 22.7°C at 8:33 to 28.8°C at 16:47. Relative humidity ranged from 37.3% at 8:41 to 31.1% at 13:44. Carbon dioxide concentrations were 550 ppm at 8:44–9:08 and 1515 ppm at 16:52 (Supplementary Table S1).

### Total Suspended Particle (TSP) Concentration

TSP concentrations determined from gravimetric analysis during the 9-h of work shift at various locations ranged 0.008–0.011 mg/m^3^. Sampling inside of the housing of the printer resulted in a non-detectable mass change on the filter concentration (limit of detection µg/m^3^) (Table 1).

**TABLE 1:** Concentrations of total suspended particulate (TSP) at various sites and particle concentration measured by DMAS, PM sensor, and OPS.

	TSP mass concentration (mg/m^3^)			
Time	Sampling time min	mg/m^3^	8 h TWA (mg/m^3^)	
Area-1	544	0.011	0.0125	
Area-2	544	0.008	0.009	
Area-3	544	0.011	0.0125	
Area-4	544	0.009	0.0102	
Area-5	-	-	-	
Inside of printer enclosure	86-	ND	ND	
Particle number concentration before exposure mitigation				
	AM		Min	Max
PM 2.5 (#cc)	0.001		0.000	0.013
DMAS (#/cc)	16,290 ± 4,468		2,569	27,005
OPS (#/L)	37 ± 7		27	72
Particle number concentration after exposure mitigation				
DMAS (#/cc)	4981		2698	7719
OPS (#/L)	71		36	185

ND, not detected; Area-1, behind of Worker 2; left side of 3-D printer-1; Area-3; top of 3-D printer A; Area-4 in front of 3-D printer-2; Area-5, Corridor; AM. Arithmetic mean; Min, minimum; Max, maximum.

### Benzene, Styrene, Total VOC (TVOC), Formaldehyde, and PAH Concentrations

Benzene and styrene measured at various time points were not detected. The TVOC concentrations ranged from 0.2 to 4 ppm with an average of 1.93 ppm. Formaldehyde at various locations was detected at 2.5–21.7 ppb, whereas the inside of the printer was 19.7 ppb, much less than ACGIH TLV-TWA (100 ppb) (2017). Total PAH levels from PAS 2000 were less than 1 ng/m^3^ throughout the measurement time (Table 2).

**TABLE 2:** Concentrations of formaldehyde before and after *via* installation of a ventilated enclosure.

	Formaldehyde (ppb)					
	Before exposure mitigation				After exposure mitigation	
Time	8:30–11:50	13:30–16:00	16:20–17:40	Time	9:00–13:00	13:00–15:00
Area-1	10.2	16.0	9.2	Area-6	23.1	9.2
Area-2	9.4	16.8	18.6	Area-7	26.3	18.6
Area-3	7.9	13.7	21.7	-		-
						-
Area-4	8.8	13.2	20.9	-	-	-
Area-5	4.6	2.5	-	Area-9	42.6	11.2
Inside of printer enclosure	-	-	19.7	Inside of printer booth enclosure (Area-8)	25.9	11.7

Outdoor formaldehyde concentration during 9:00–15:30 was 11.5 ppb.

### Concentrations of VOCs by PTR-TOF-MS

A wide variety of harmful gases were detected at low concentrations down to the ppb level (Table 3). However, acetone was at a high ppm concentration because the surface of ABS products was intermittently wiped with acetone. Even though no wiping of ABS surfaces was conducted on the day of this measurement, it is believed that some acetone was residue leftover from previous work. The sum of individual VOCs was 2.085 ppb, which was similar to the average 1.93 ppb result by the TVOC sensor.

**TABLE 3:** Concentrations of VOCs by PTR-TOF-MS.

VOCs	Mean	S.D.	Min	Max
Formaldehyde	29.8	1.8	26.9	33.4
Acetaldehyde	8.4	0.2	8.0	8.8
Acetone	1493.6	18.5	1461.4	1540.8
Acrolein	6.3	0.8	5.4	10.7
Benzene	0.4	0.0	0.4	0.4
1,3-Butadiene	0.2	0.0	0.1	0.2
Chloroethylene	1.1	0.1	1.0	1.2
Chloroform	3.3	0.1	3.0	3.5
Dimethyl disulfide	2.4	0.1	2.1	2.7
Ethanol	181.5	7.2	174.2	214.5
Ethylbenzene	1.3	0.1	1.2	1.4
Hexane	10.6	0.5	9.9	12.2
Isopropyl Alcohol	66.3	5.3	58.4	78.2
Methylethylketone	4.3	0.4	3.5	4.8
Methanol	102.5	0.9	100.3	104.4
PGME^a^	9.4	0.6	8.0	11.0
Phenol	8.2	0.6	6.7	9.6
Propene	124.9	10.3	109.4	147.3
Styrene	2.7	0.3	2.4	3.6
Toluene	13.0	0.4	12.4	14.2
Trimethylamine	13.1	0.8	11.8	15.3
Xylene	1.3	0.1	1.2	1.4

Unit: ppb

a Propylene glycol monomethyl ether.

### Real-Time Monitoring of Particle Measurement and Formaldehyde Concentration Before and After Enclosure

**FIGURE 2:**
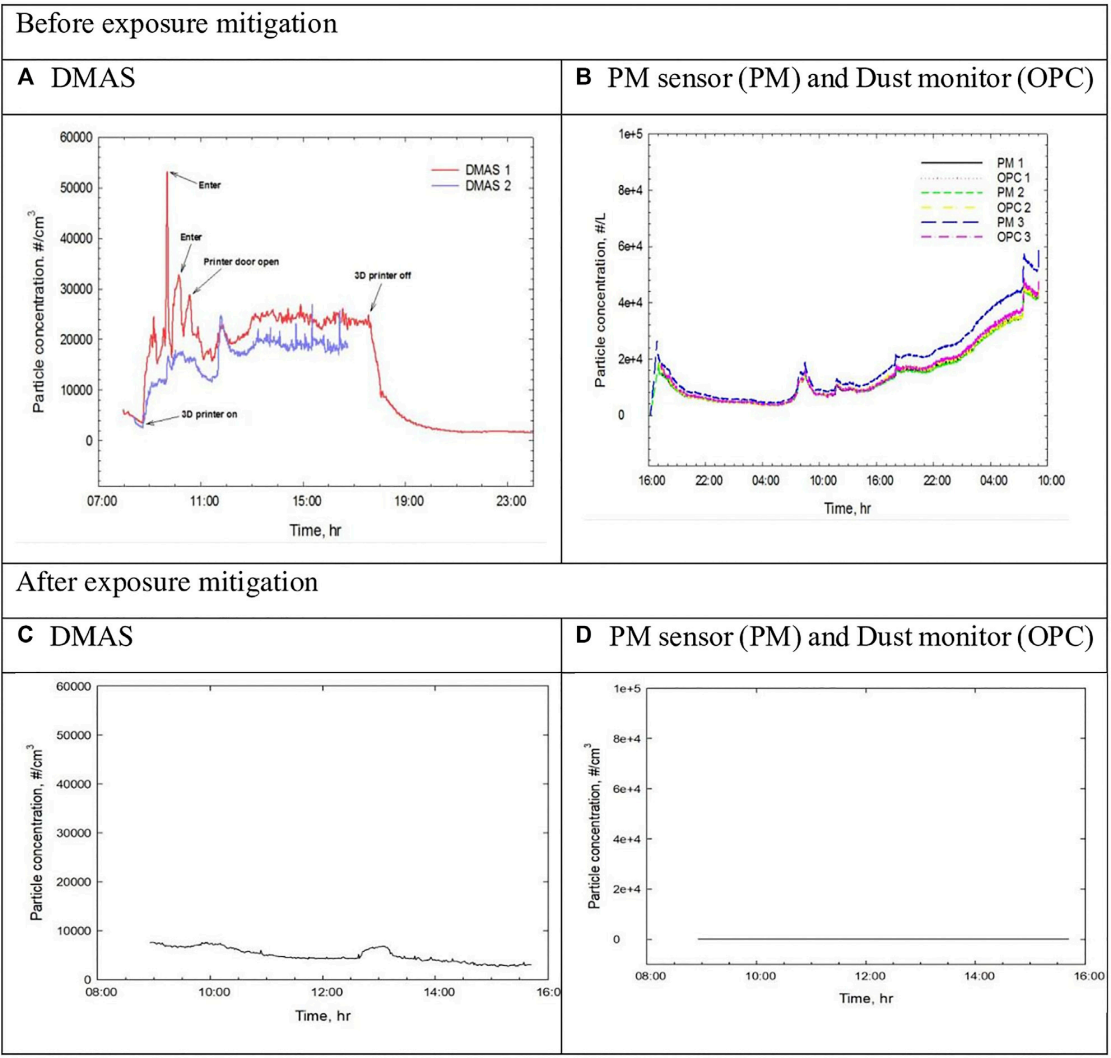
Real-time particle measurement in 3-D printing workplace. **(A)**, DMAS; **(B)**, PM sensor (PM) and Dust monitor (OPC).

UFPs measured by DMAS ranged between 2,569–27,005 particle/cm^3^ with an average of 16,290 ± 4,468 particle/cm^3^ during the operation of 3-D printers (Table 1). After switching on the printers, particle number concentration began to increase. When the printers were turned off at 6 PM, the numbers decreased to less than 2000 (Figure 2A). On the other hand, fine particle numbers measured from 16:00 on the day before 3-D printer operation to 8:00 on the day after printer operation showed a different pattern from DMAS measurement. Before starting 3-D printers, fine particle concentration showed 2000–3000 particle numbers/liter and reached approximately 18,000 particle/liter at the start of 3-D printers and continuously increased to 40,000–50,000 particles/liter until the next day morning (Figure 2B). Particle number measurement conducted inside the 3-D printer enclosure resulted in no data. It could be caused by a high-temperature process affecting DMAS measurement or not measurable size by DMAS due to the vapor state of the emission. Our result indicates that the UFPs (∼30 nm) emitted from 3-D printers slowly transformed into fine particles larger than 200 nm (Supplementary Figure S1).

After the initial exposure assessment, the management of the workplace noticed the status of exposure to the 3-D printer emission and initiated exposure mitigation. They isolated the two 3-D printers in the enclosed space, which has ventilation on the ceiling (Figure 1B). The nanoscale particles originating from the 3-D printers were greatly reduced after mitigation measure (Figure 2C), while particles detected by OPC increased little (Figure 2D). After the mitigation measure, formaldehyde at various locations described in Figure 1B ranged 23.1–42.6 ppb at 9:00–13:00 and 9.2–18.6 ppb at 13:00–15:00. The higher concentrations of formaldehyde in the morning time could be caused by floor cleaning and waxing, which may use formaldehyde-containing agents, while the concentrations were low in the afternoon (Table 2).

### The Size Distribution and MMAD of Ultrafine Particles

**FIGURE 3:**
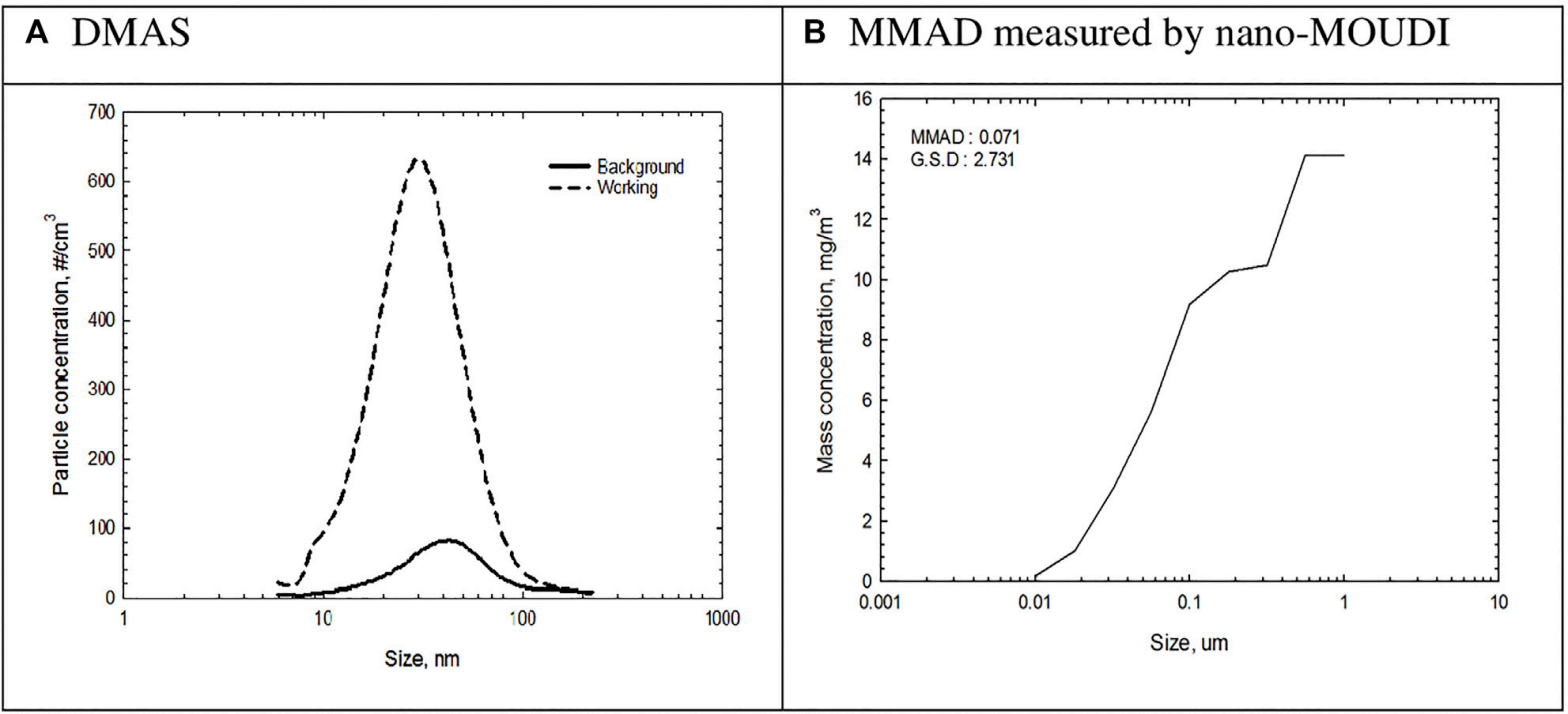
Size distribution of 3-D printer emissions measured by DMAS and Nano-MOUDI. **(A)** Result of DMAS, **(B)** Result of median mass aerodynamic diameter (MMAD) by nano-MOUDI.

Count median diameter (CMD) measured by DMAS was 30 nm (Figure 3A), and MMAD and GSD measured by nano-MOUDI were 71 nm and 2.731, respectively (Figure 3B). Size distribution measured by dust monitors showed the formation of slightly smaller particles compared to the background (Supplementary Figure S2).

### TEM Analysis

**FIGURE 4:**
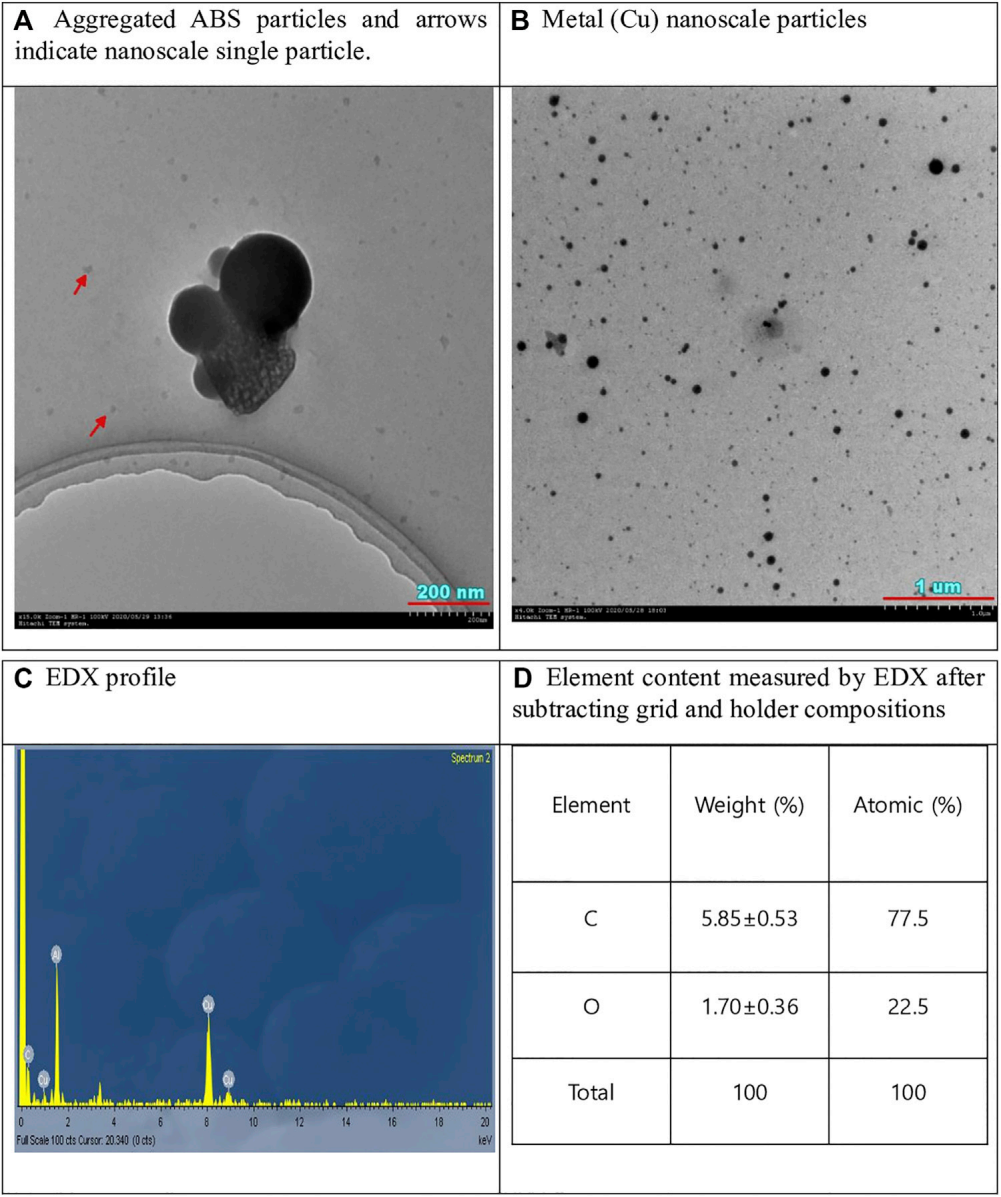
TEM micrograph of ABS particles and EDX-analysis.

Well dispersed ABS particles were observed with TEM, where some particles were aggregated (Figures 4A,B). EDX analysis indicated that most particles consisted of carbon when subtracting Cu and Ni as grid and Al as holder compositions (Figure 4D). Unexpectedly, copper metal nanoparticles were also observed, which are believed to be impurities added (pigments, etc.) to ABS materials. The ABS filament was further analyzed by ICP-MS, and the results showed that ABS filament contained some impurity metals (Al 62 ppm; Cr 2.6 ppm; Fe 13 ppm; Cu 1.1 ppm). However, metal nanoparticles were observed only in some areas of the grid, whereas carbon ABS nanoparticles were evenly distributed in a wide area of the grid.

## Discussion

This study has conducted a comprehensive characterization of exposures, including VOCs, particle number and mass concentration, size distribution, and elemental composition/morphology. Exposure assessment documented low levels of total suspended particulates (9–12.5 µg/m^3^), minimal levels (1.93–4 ppm) of TVOC as measured by real-time monitor, and formaldehyde (2.5–21.7 ppb), with no detectable levels of benzene and styrene by GC-FID. However, PTR-TOF-MS analysis of grab samples detected various chemicals at low concentrations, the majority of which were in the low ppb. The reason is that PTR-TOF-MS has much better analysis sensitivity than GC/FID. UFPs emitted from 3-D printers had an average particle size of 30 nm CMD and 71 nm MMAD, and their size continued to increase after the termination of 3-D printing through the night until the next day morning. The particle number concentration reduced greatly after installation of an ventilated enclosure for the 3-D printers.

Our particle size measurement of 3-D printing emission from the inside of the 3-D printer-1 enclosure indicated that 3-D printer-1 emissions generated as vapors of semi-volatile organics could not be measured with DMAS, and as they cooled off, the vapors condensed to form the nucleation stage, where their size is measurable by DMAS, to further coagulation stage where their size is measurable by OPC or dust monitor. In contrast, emissions taken from 3-D printer-2 showed 1.73-2.50 × 106 particle/cc depending on the 3-D printing process. Our UFP concentration of 16,000 particle/cc during printer operations was similar to the range reported by other studies (Stephens et al., 2013; Afshar-Mohajer et al., 2015; Deng et al., 2016; Steinle, 2016; Mendes et al., 2017; Vance et al., 2017). Total VOC (TVOCs) concentrations from 3-D printing emission were also similar to concentrations of 102–103 μg/m^3^ reported previously (Azimi et al., 2016; Steinle, 2016; Floyd et al., 2017; Mendes et al., 2017).

Potential health effects of the particulates and VOC compounds emitted by 3D printers were studied by various authors using either acellular or *in vitro* cellular toxicity testing systems. These early results indicated that ABS, polycarbonate (PC), and polyacrylic filament emissions induced significant dose-dependent cytotoxicity, oxidative stress, apoptosis, necrosis, and inflammation, documented by the production of several key pro-inflammatory cytokines (Farcas et al., 2019; Zhang et al., 2019)]. In an experimental 1-h exposure study of healthy human volunteers to ABS- and PLS- based 3-D printer emissions, no significant effects were seen on 8-iso PGF2α and nasal biomarkers such as IL-1, IL-6, TNF-α, and IFN-γ. However, there was a statistically significant difference (*p* < 0.05) in the time course of exhaled nitric oxide, with higher, exhaled nitric oxide levels (19.1 ppb) measured following ABS exposure (1 × 10^6^/cc). Moreover, the authors of the study suggested that the slight relative increase in exhaled nitric oxide after ABS exposure compared to PLA might be due to eosinophilic inflammation from inhaled UFPs (Gümperlein et al., 2018). Further studies are needed to better assess potential health effects of 3-D printer emissions.

There are several exposure mitigation strategies in the workplace; *1*) elimination of the hazard or substitution of hazard materials, *2*) engineering control including enclosure or encapsulation which isolates emission source, or installing local ventilation or general ventilation to reduce exposure to emission, *3*) administrative control reducing the duration of exposure by limiting working hour, and *4*) use of personal protective equipment (PPE) such as mask or respirator. Among them, the engineering control, including enclosure or encapsulation, which isolates emission source, is the second-best exposure mitigation. Our exposure assessment result after mitigation measure clearly indicated the reduction of 3-D emission. The concentration of formaldehyde monomer suspected to be released during 3-D printing from ABS polymer ranged 26.8–33.4 ppb with an average of 29.8 ppb. After mitigation of exposure, formaldehyde concentration 23.1–26.3 ppb with an average of 24.7 ppb in the morning, presumably concentration was affected by cleaning and waxing of floor in the morning, and 9.2–18.6 ppb with an average of 13.9 ppb in the afternoon, indicating a reduction of VOC after mitigation.

## Conclusion

A comprehensive characterization of exposures to 3-D printer emission including VOCs, particle number concentration, mass concentration, size distribution, and elemental composition/morphology resulted in low levels of total suspended particulates (9–12.5 µg/m^3^), minimal levels (1.93–4 ppm) of TVOC, and formaldehyde (2.5–21.7 ppb), with no detectable levels of benzene and styrene by GC-FID. PTR-TOF-MS analysis of grab samples detected various chemicals at low concentrations, most of which were in the low ppb. UFPs emitted from 3-D printers had an average particle size of 30 nm CMD and 71 nm MMAD, and their size continued to increase after the termination of 3-D printing through the night until the next morning. After recognizing emissions from 3-D printers, the workplace initiated 3-D printer emission exposure mitigation by encapsulating the 3-D printers. After mitigation, the exposure assessment showed a reduction of 3-D printer emissions and some indication of VOC reduction, as indicated by VOC reduction indicated by formaldehyde concentration.

## Data Availability

The original contributions presented in the study are included in the article/Supplementary Material, further inquiries can be directed to the corresponding authors.
