# Supplementary material for: Assessment and Mitigation of Exposure of 3-D Printer Emissions
Source: Front Toxicol. 2022 Feb 18;3:817454. doi: 10.3389/ftox.2021.817454 (PMC8915804; doi:10.3389/ftox.2021.817454)
Supplement: Supplementary file 1 [file DataSheet1.docx]

Table S1. Time course events for 3-D printing workplace. Activities during 3-days of monitoring were recorded with temperature, humidity and CO_2_ concentration.

| Day | Time | Operation | Temperature, Humidity, CO_2_ |
| --- | --- | --- | --- |
| 1st day | 17:00 | Start of PM sensor measurement |  |
| 2nd day | 7:58 | Start of DMAS measurement |  |
|  | 8:31 | Pump sampling start |  |
|  | 8:31 | 3-D print start | 22.7^o^C |
|  | 8:41 | Door closed | 37.3% |
|  | 8:44 ~ 9:08 | 4 times door open | 530 ppm |
|  | 9:09 | DMAS port change of location |  |
|  | 9:11 ~ 9:35 | 3 times door open |  |
|  | 9:42 | Printer-2 door open |  |
|  | 9:43 | Door open |  |
|  | 9:45~9:48 | Printer-2 door open |  |
|  | 9:51 | Door open, TEM sampling, Printer-2 door open |  |
|  | 9:52 | Printer-2 door open |  |
|  | 9:53 | Door open |  |
|  | 9:55 | Door open, Printer-1 door open |  |
|  | 9:56 | Printer-2 door open |  |
|  | 10:03 | Printer-2 door open |  |
|  | 10:07 ~ 10:13 | 3 times door open |  |
|  | 10:14 | 2 times printer-2 door open |  |
|  | 10:15 | printer-2 stop |  |
|  | 10:18 | Door open |  |
|  | 10:20 | printer-2 restart |  |
|  | 10:21 | printer-2 door open |  |
|  | 10:23 ~ 10:34 | 4 times door open |  |
|  | 10:35 | printer-2 door open |  |
|  | 10:47 | printer-2 door open |  |
|  | 11:09 ~ 11:25 | 3 times door open |  |
|  | 11:26 | printer-2 door open |  |
|  | 11:27 ~ 11:38 | 3 times door open |  |
|  | 11:41 | MOUDI sampling pump box open |  |
|  | 11:44 ~ 12:01 | 8 times door open |  |
|  | 12:01 ~ 13:00 | Lunch time |  |
|  | 13:02 | MOUDI sampling start |  |
|  | 13:05~13:31 | 6 times door open |  |
|  | 13:32 | printer-2 door open |  |
|  | 13:33 ~ 13:35 | 3 times door open |  |
|  | 13:38 | Robot start |  |
|  | 13:44 ~ 13:58 | 2 times door open | 31.1% |
|  | 14:02 | MOUDI turn off |  |
|  | 14:13 ~ 14:55 | 5 times door open |  |
|  | 14:59 | printer-2 door open |  |
|  | 15:01 | printer-2 door open |  |
|  | 15:17 | TEM sampling (2 min) |  |
|  | 15:18 | MOUDI sampling start in 3-D printer |  |
|  | 15:48 | MOUDI turn off |  |
|  | 16:16 | Printer-1 door open, |  |
|  | 16:29 | Printer-1 door open |  |
|  | 16:47 | Printer-2 door open | 28.8^o^C |
|  | 16:52 | DMAS connected to inside of printer | 1515 ppm |
|  | 17:34 | Printer-1, 2 turn off |  |
|  | 17:37 | Area-1,2,3 pump turn off |  |
|  | 17:39 | Take out a printout of printer-2 |  |
|  | 17:42 | Take out a printout of printer-1 |  |
| 3rd day | 9:16 | PM sensor, DMAS turn off |  |

Supplement

Figure S1. 3-D printed objects.

| A. Printer 1 | B. Printer 2 |
| --- | --- |
| 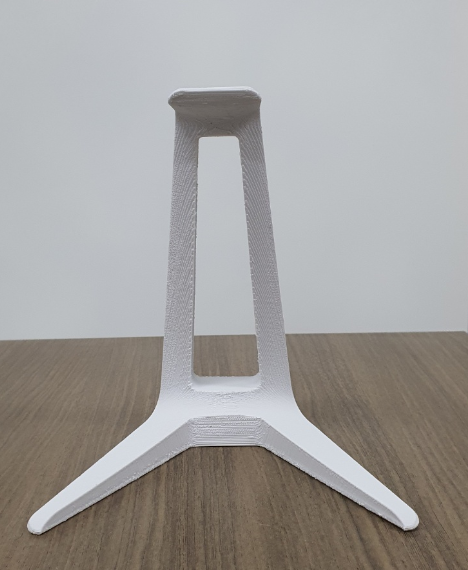 | 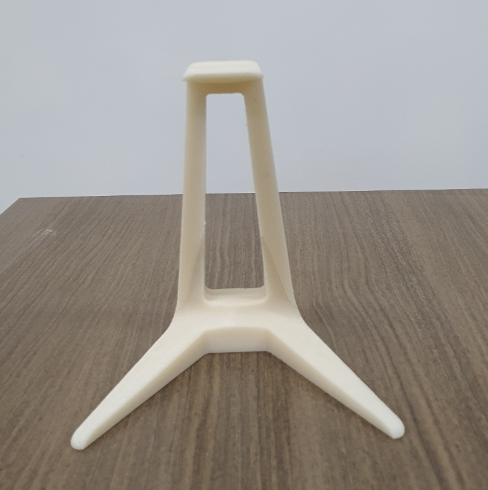 |

Figure S2. Size distribution measured by Dust monitor. A, Dust monitor 1; B, Dust monitor 2; C, Dust monitor 3.

| A. Dust monitor 1 | B. Dust monitor 2 | C. Dust monitor 3 |
| --- | --- | --- |
| 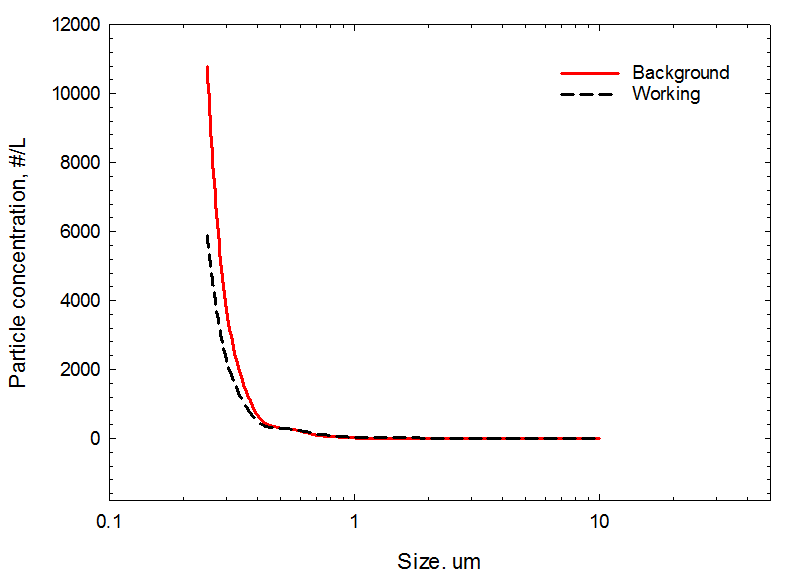 | 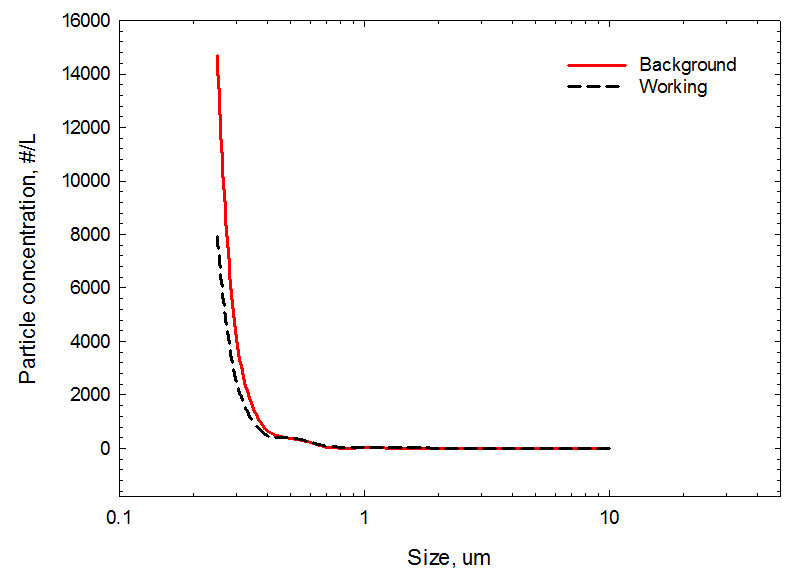 | 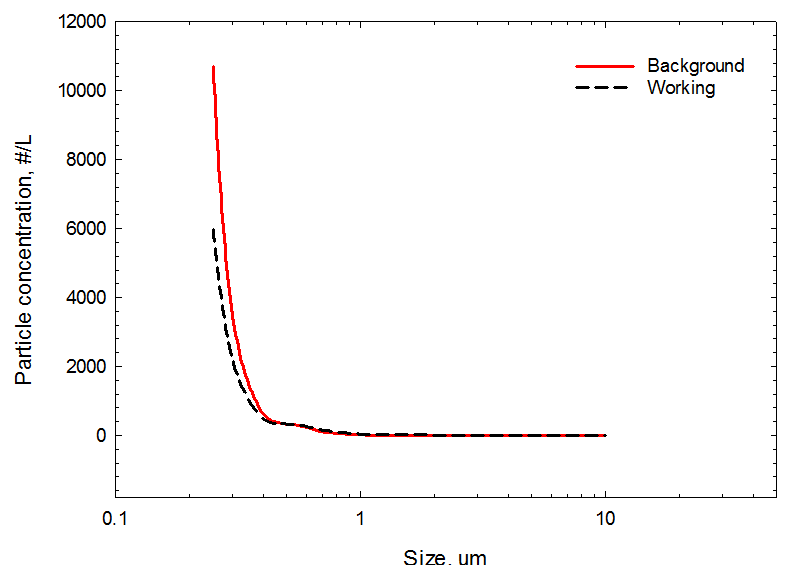 |
